# A Lightweight Hybrid Detection System Based on the OpenMV Vision Module for an Embedded Transportation Vehicle

**DOI:** 10.3390/s25185724

**Published:** 2025-09-13

**Authors:** Xinxin Wang, Hongfei Gao, Xiaokai Ma, Lijun Wang

**Affiliations:** Department of Mechanical Engineering, North China University of Water Resources and Electric Power, Zhengzhou 450000, China; wangxinxin@ncwu.edu.cn (X.W.); z20241040479@stu.ncwu.edu.cn (X.M.); wljmb@163.com (L.W.)

**Keywords:** mobile embedded devices, object detection, OpenMV vision module, FOMO MobileNetV2 model, lightweight hybrid detection system

## Abstract

Aiming at the real-time object detection requirements of the intelligent control system for laboratory item transportation in mobile embedded unmanned vehicles, this paper proposes a lightweight hybrid detection system based on the OpenMV vision module. The system adopts a two-stage detection mechanism: in long-distance scenarios (>32 cm), fast target positioning is achieved through red threshold segmentation based on the HSV(Hue, Saturation, Value) color space; when in close range (≤32 cm), it switches to a lightweight deep learning model for fine-grained recognition to reduce invalid computations. By integrating the MobileNetV2 backbone network with the FOMO (Fast Object Matching and Occlusion) object detection algorithm, the FOMO MobileNetV2 model is constructed, achieving an average classification accuracy of 94.1% on a self-built multi-dimensional dataset (including two variables of light intensity and object distance, with 820 samples), which is a 26.5% improvement over the baseline MobileNetV2. In terms of hardware, multiple functional components are integrated: OLED display, Bluetooth communication unit, ultrasonic sensor, OpenMV H7 Plus camera, and servo pan-tilt. Target tracking is realized through the PID control algorithm, and finally, the embedded terminal achieves a real-time processing performance of 55 fps. Experimental results show that the system can effectively and in real-time identify and track the detection targets set in the laboratory. The designed unmanned vehicle system provides a practical solution for the automated and low-power transportation of small items in the laboratory environment.

## 1. Introduction

The design of vision-based control systems for autonomous vehicles is currently a major research focus in the transportation domain and represents a key developmental trend for future transportation technology [1]. In terms of hardware, the primary approach involves a fusion of various sensors, including LiDAR, cameras, and millimeter-wave radar [2]. For instance, Lu Y. W., Tang W. T. et al. [3] proposed an automatic extrinsic calibration method combining LiDAR’s non-repetitive scanning pattern with cameras. This method utilizes a specially designed perforated checkerboard calibration target, extracting and matching geometric features from both point clouds and images. Compared to traditional methods, it demonstrates significant advantages in calibration accuracy, efficiency, and applicability.

Regarding software research, efforts both domestically and internationally primarily concentrate on computer vision algorithms and deep learning techniques. For example, Zhou, Z.X., Song, Z.Z., et al. [4] developed an Android application named KiwiDetector for kiwifruit detection in orchards. They employed a Single Shot Multibox Detector (SSD) framework using two lightweight backbone networks: MobileNetV2 and InceptionV3. To compress the model size and improve detection speed, they implemented an 8-bit quantization method, converting the convolutional neural network’s weight tensors and activation function data from 32-bit floating-point format to 8-bit integers. This significantly reduced the computational load. Zhang X.F. [5] designed a dynamic visual SLAM system based on RGB-D cameras. Specifically, the system employs the SegNet network for semantic segmentation and integrates the YOLOv5 model for object detection, enabling accurate identification of potential moving objects while ensuring real-time performance.

LiDAR is relatively sensitive to environmental conditions and has high requirements for computational resources and power consumption; traditional machine vision detection methods are costly, mainly used in intelligent driving vehicles and intelligent robots, but not suitable for small mobile embedded unmanned transport vehicles [6]. The color threshold-based methods for traditional robots and embedded devices have poor robustness under complex lighting and high false detection rates; while deep learning models improve accuracy, their computational complexity hardly meets the real-time requirements of embedded terminals [7]. Therefore, this paper proposes a lightweight hybrid detection system based on the OpenMV vision module. The system adopts a two-stage detection strategy and a heterogeneous computing architecture. When the distance is greater than 32 cm, HSV(Hue, Saturation, Value) color space threshold segmentation is enabled for fast rough positioning, and when the distance is less than or equal to 32 cm, it switches to the FOMO MobileNetV2 lightweight model for fine-grained classification. This “perception, decision, and control” closed-loop architecture has been successfully deployed on a small-scale laboratory unmanned goods transport vehicle platform equipped with the STM32H7 processor, providing a cost-effective environmental perception solution for resource-constrained mobile devices.

## 2. Hardware and System Design

The STM32F103C8T6 microcontroller (STM32F103C8T6, STMicroelectronics, Geneva, Switzerland) adopts an LQFP48 package, enabling it to rapidly process complex control tasks and provide strong peripheral support [8]. The STC series microcontrollers support serial port programming, featuring low usage costs that significantly shorten development time. Peripheral devices include an OLED display, Bluetooth module, ultrasonic module, motor module, OpenMV camera, TTL to USB serial port module, STLINK programmer, external power supply, and corresponding voltage regulator modules.

The body shell of the system is primarily constructed from acrylic panels (PMMA), with main connections realized through screws and nuts. Copper pillars provide critical support, height adjustment, and isolation for electronic components. The microcontroller, serving as the core control unit, along with various sensors, actuators, and a breadboard for circuit prototyping, is meticulously integrated and firmly mounted within the internal structure of the body shell. To ensure the safety of the entire system during storage and transportation, a transport case made of PVDF (polyvinylidene fluoride) is equipped. This case features excellent chemical stability (resistant to strong acids, alkalis, and organic solvents), a wide temperature tolerance range (−40 °C to 150 °C), and lightweight characteristics [9], providing optimal protection for laboratory item transportation.

**Figure 1:**
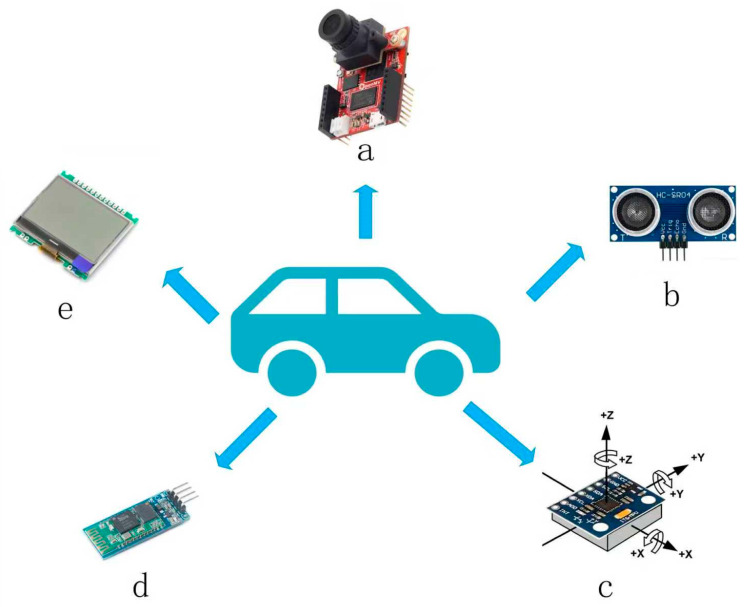
Diagram of the multimodal perception system, where (a) is the OpenMV vision module, (b) is the ultrasonic module, (c) is the MPU6050 attitude correction unit, (d) is the Bluetooth module, and (e) is the OLED display system.

The cart designed in this paper integrates multimodal perception and intelligent control functions, as shown in Figure 1. Each module is presented, with the technical characteristics of each module as follows:

- OLED Display System: Equipped with a high-contrast OLED display, it visualizes in real time motion parameters, sensor data, and functional status, constructing an intuitive human–machine interaction interface.
- Ultrasonic Obstacle Avoidance System: Featuring an HC-SR04 ultrasonic module, it scans a 180° fan-shaped area to real-time detect obstacles within 0.02–4 m. Combined with dynamic obstacle avoidance algorithms, it automatically plans safe paths, enhancing operational safety in complex environments.
- MPU6050 Attitude Correction Unit: Integrating a six-axis inertial measurement unit, it collects real-time three-axis acceleration and angular velocity data, realizing motion attitude calculation via complementary filtering algorithms. It has dynamic trajectory correction capability: when external forces cause heading deviation, it completes attitude calibration and returns to the preset route within 200 ms, while supporting 0–360° precise angle rotation control.
- Bluetooth Wireless Control Module: Using the BLE 5.0 communication protocol, it establishes a 10 m radius wireless control range, enabling experimenters to remotely control basic actions of the cart (start/stop, speed adjustment, steering) via a mobile APP.
- OpenMV Visual Navigation System: Equipped with an OV5640 camera (OV5640, OmniVision Technologies, Inc., Santa Clara, CA, USA), it performs real-time recognition and positioning of red-marked target areas based on machine vision algorithms. The STM32 main control chip processes image data and generates motion control commands, constructing a “visual perception-decision control” closed-loop system that supports precise target area arrival and dynamic following in cargo transportation scenarios.

### 2.1. Speed and Mileage Display of DC Gear Reduction Encoder Motor and OLED Screen

The drive motor selected in this article is the JGA25-370 DC geared encoder motor, a type of DC gear motor with an encoder. The Hall encoder, composed of a Hall code disc and Hall elements, serves as an integral component of DC geared motors. As a sensor, it converts the mechanical geometric displacement of the output shaft into pulse or digital signals through electromagnetic conversion. This conversion enables precise measurement of rotational position and speed, making it critical for closed-loop speed control systems [10].

As shown in Formula (1), the method of measuring real-time speed using the pulse frequency method is shown [11].

(1) $$\omega =(2\pi \cdot f_{\mathrm{pulse}})/N_{\mathrm{res}}\;\;\;\;[rad/s]$$

ω is the angular velocity of the motor shaft (rad/s);

$f_{pulse}$ is the pulse frequency (Hz), i.e., the number of pulses per unit time;

$N_{res}$ is the Resolution (PPR, Pulses Per Revolution).

The OLED module is used for displaying numerals, characters, and text with high contrast [12]. The pin configurations are as follows: GND: Ground connection for electrical potential reference; VCC: 5 V power supply input; SCL (Serial Clock Line): Connected to the PA15 port of the STM32 microcontroller; SDA (Serial Data Line): Connected to the PB12 port of the STM32 microcontroller. The Hall encoder collects speed data and transmits it to the STM32 microcontroller. The microcontroller encodes and processes the data, enabling the speed information to be displayed on the OLED.

Based on the calculated motor speed, the total mileage is obtained by accumulating the driving distances within each unit time interval. In the paper, the driving distance is calculated every 20 milliseconds (ms) and continuously summed up. Given a wheel diameter of 7 cm, the circumference is C=Π×d=3.14×4≈22 cm. If the motor speed is in revolutions per second, the distance traveled in 20 ms is expressed as 0.02×Motor1speed×22 cm. By continuously accumulating the distances calculated for each time interval, the total driving mileage of the cart is obtained. This method ensures the cumulative accuracy of mileage data through precise time segmentation and geometric parameter calculations of the wheels.

### 2.2. MPU6050 Six-Axis Sensor Attitude Correction Technology

This module is used to prevent the trajectory deviation of the car when it collides with an external force. The gyroscope sensor can detect the change of angle in real time, but using the gyroscope will accumulate errors, so it also needs the acceleration sensor to collect the angular velocity to correct them, and this is the advantage of MPU6050. Its communication mode is the I2C communication protocol, which makes it convenient to access the acquisition information output through the serial port. The module also has a filter circuit, which can process the noise signal, so as to make the detected signal more accurate [13].

The angular velocity measurement formula of the gyroscope is as shown in (2).

(2) $$\omega_{gyro}=\frac{d\theta }{dt}+\varepsilon_{bias}+\varepsilon_{noise}$$

$\omega_{gyro}$: Instantaneous angular velocity of the gyroscope;

θ: True rotation angle;

$\varepsilon_{bias}$: Bias drift error (accumulates over time);

$\varepsilon_{noise}$: High-frequency measurement noise.

Accelerometer Tilt Angle Calculation:

Static Tilt Angle Calculation via Gravitational Component Decomposition (insensitive to cumulative errors), as shown in Formulas (3) and (4).

(3) $$\theta_{acc}=\arctan\left(\frac{a_{x}}{a_{z}}\right)$$

(4) $$\theta_{acc}=\arctan\left(\frac{a_{y}}{a_{z}}\right)$$

$a_{x},\;a_{y},\;a_{z}$: Raw data from a three-axis accelerometer (m/s^2^).

Limitations: Motion acceleration during vehicle acceleration/deceleration will interfere with the measurement of gravitational components.

The track deviation judgment formula is shown in (5).

(5) $$y_{offset}=\int \left(v\times \sin\left(\Delta \theta \right)\right)dt$$

v: Vehicle speed (acquired via wheel speed sensor);

Δθ: Fused actual yaw angle (°);

Judgment criterion: Initiate deviation correction control when $\left|y_{offset}\right|>\;y_{threshold}$.

In this paper, vehicle angle data are collected in real time by sensors, and a set of PID parameters for attitude control is defined in the program. The PID parameters set in this paper are adjusted by experience through iterative testing in a controlled environment. The sign and magnitude of the proportional gain (P) are first adjusted, followed by the sign of the derivative gain (D) in sequence. The final tuning effect demonstrates that when the vehicle is placed on the ground, it maintains the initial heading direction upon power-up; if pushed laterally, it immediately corrects its course. The specific parameter definitions are shown in Table 1.

### 2.3. Ultrasonic Module HC-SR04 and Mobile Phone Remote Control

The ultrasonic distance measurement circuit designed in this paper employs the off-the-shelf HC-SR04 ultrasonic module, which enables non-contact distance measurement within the range of 2–500 cm with a precision of up to 3 cm [14]. The operation process starts with the initialization of module pins, then acquires front distance data and sends the distance information to the STM32F103C8T6 microcontroller in real time. When the distance to the target is within the set range, the STM32F103C8T6 microcontroller controls the motor to perform obstacle avoidance actions, while transmitting distance data to the OpenMV vision module via serial communication to assist it in initiating target detection within the specified distance.

**Figure 2:**
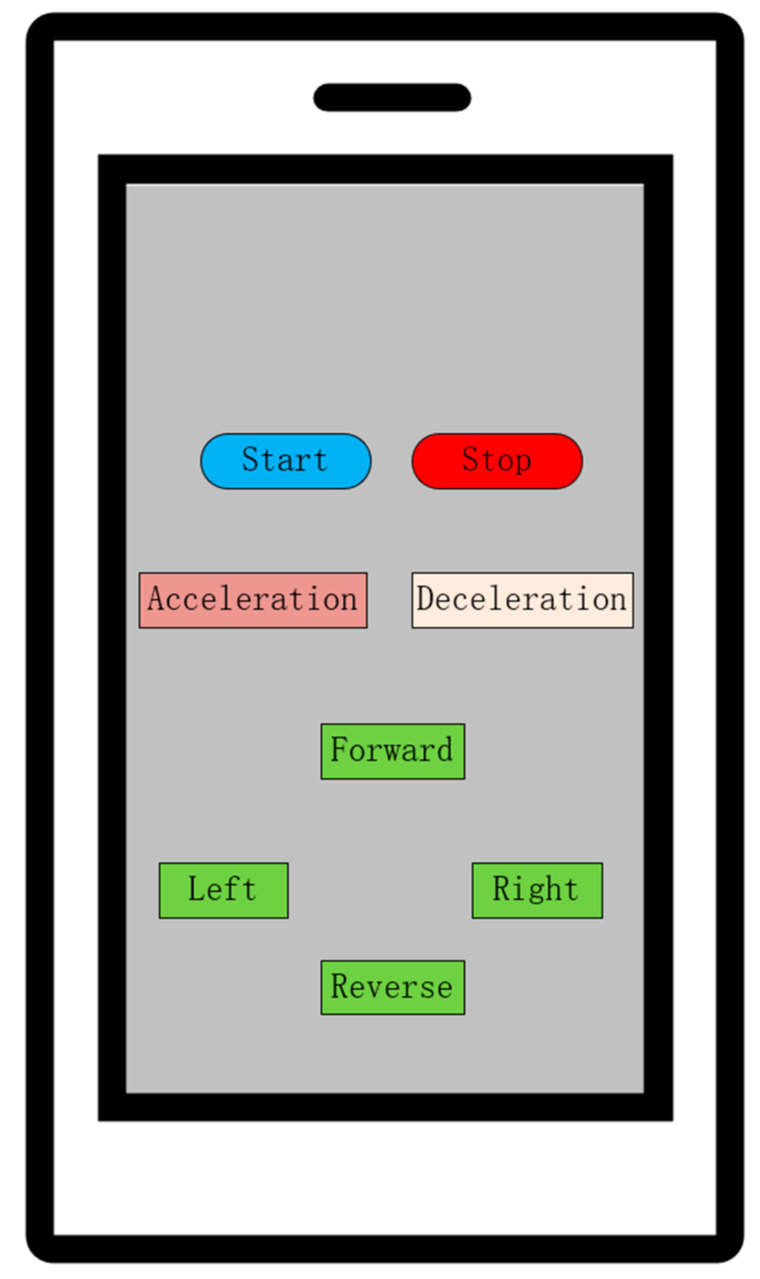
Mobile phone APP remote control interface.

The HC-05 Bluetooth module is a serial communication transmission device specifically designed for short-range wireless communication. It features high sensitivity, ease of development, high cost-effectiveness, and flexible deployment [15,16]. To realize the Bluetooth remote control function, the serial port of the microcontroller and the Bluetooth communication module are required. The debugging process first involves establishing a connection between the microcontroller and the Bluetooth module via the serial port, as well as a connection between the Bluetooth module and the mobile phone, followed by integrating these two communication links. In this paper, a Bluetooth debugger APP is used to achieve mobile phone remote control, and its control interface is shown in Figure 2. During debugging, the pairing between the Bluetooth module and the mobile phone is first completed; after pressing the “Start” button on the control interface, the car enters the state of receiving data from the mobile phone; when the “Forward” button is pressed, the mobile phone transmits commands to the Bluetooth module via Bluetooth, which then forwards them to the microcontroller to drive the motor. Ultimately, the seamless transmission of remote control commands from the mobile device to the microcontroller drive system is achieved.

## 3. Experiment and Verification

### 3.1. A4950 Motor Drive and PWM

The A4950 motor (A4950, Allegro MicroSystems, Inc., Manchester, NH, USA) driver module boasts advantages such as large driving current, wide voltage range, and microcontroller resource savings, requiring only two PWM signals to control a single motor. PWM technology plays an important role in the system design process, primarily serving to control motor speed. This paper adopts a combined approach of high–low level direction control and PWM speed regulation, achieving motor control while saving microcontroller resources [17].

**Figure 3:**
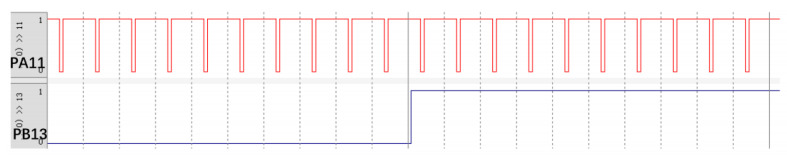
PWM simulation diagram: PA11 pin outputs 90% duty cycle, Pb13 pin outputs high level first, and then Pb13 pin outputs low level.

The AIN2 and BIN2 pins of the A4950 driver board control the speed by regulating the outputs of timers PWMA and PWMB, and are connected to the PA11 and PA8 pins of the microcontroller. The control principle is as follows:

The PWM simulation is shown in Figure 3. Using PA11 and PB13 to control motor 1: When PA11 (AIN2) outputs a 90% duty cycle and PB13 (AIN1) is at a low level, the motor rotates forward, with the speed corresponding to 90% of the voltage. When PA11 (AIN2) maintains a 90% duty cycle and PB13 (AIN1) is at a high level, the motor rotates in reverse, and the speed corresponds to 10% of the voltage value (since the actual speed is determined by the duty cycle difference between the two PWM signals, i.e., 100 − 90% = 10%).

### 3.2. Motor Speed and Direction Control

**Figure 4:**
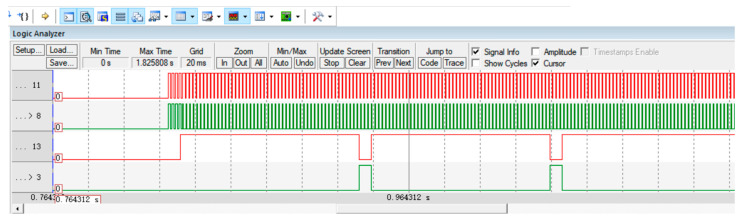
PWM simulation diagram: PA11 and PA8 pins output PWM signals to control motor speed, and Pb13 and PB3 pins output high and low levels to control motor direction.

After setting up the PWM output, it is necessary to design and program the motor control code. To shorten debugging time and avoid errors in actual operation, a logic analyzer was used for software simulation. When writing the motor control code, it should be noted that the two pairs of motors are installed in reverse; therefore, the levels for controlling forward and backward movement are also opposite. The PWM simulation is shown in Figure 4.

A positive value for the motor indicates forward rotation, while a negative value indicates reverse rotation or backward movement. The absolute value of the motor command must not exceed the PWM amplitude. When the motor value is negative and its magnitude exceeds the PWM amplitude, the duty cycle is calculated as 100% + (current duty cycle), which corresponds to the motor speed.

Test results show that the right wheel of the actual vehicle rotates backward at a speed of 2 revolutions per second, while the left wheel rotates forward at 2 revolutions per second.

### 3.3. Debugging Experiment of Speed Control

Speed control is crucial during the operation of the car. Without PID control, the car’s operation would encounter problems such as jitter, instability, and insensitivity. Furthermore, without PID, the car cannot quickly reach the set speed value. All these issues would cause the car to fail to operate normally, thereby failing to achieve the ultimate goal.

**Figure 5:**
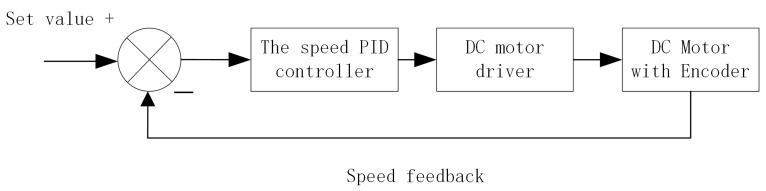
Block diagram of speed closed-loop energy control.

This paper adopts speed closed-loop control, also known as incremental PID control, which is different from positional PID control. Positional PID control outputs a new state of the control variable by calculating the error, while incremental PID outputs the increment of the control variable. Speed closed-loop control aims to make the motor operate at a specific speed [18]. Its control block diagram is illustrated in Figure 5.

**Figure 6:**
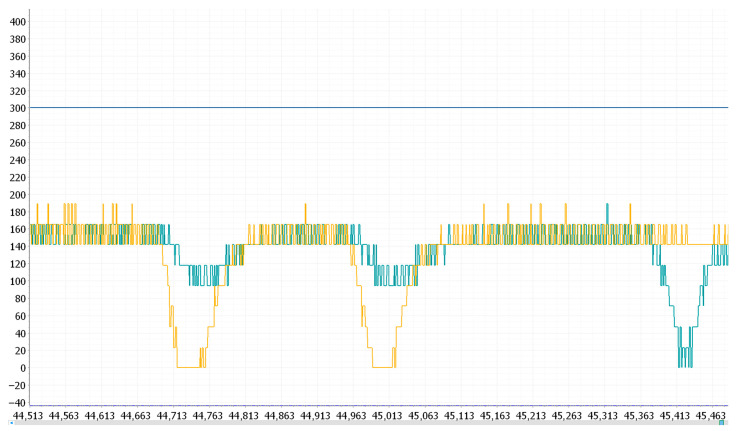
Motor speed waveform of the anonymous host computer. Yellow and green are motor speed waveforms. When resistance is applied, the motor speed fluctuates and returns to normal after a period of adjustment.

Previously, speed could be measured via the encoder, thus enabling speed control. In the program, we only need to write code to maintain the rotational speed between 2.9 and 3.1 revolutions per minute (RPM). The observed phenomena are as follows:

The motor initially failed to reach 3 RPM but gradually met the requirement as the PWM duty cycle increased. After reaching the target speed, when resistance was applied to the motor, the motor speed decreased; after a period of adjustment, the speed returned to the target value. The observed motor speed waveform in the anonymous host computer (mainly used to communicate with slave devices such as microcontrollers, sensors, and other hardware devices to realize the function of data monitoring) is shown in Figure 6.

In the absence of a PID control algorithm, the actual rotational speed of the motor often fails to reach the designed target value due to factors such as load variations, voltage fluctuations, or frictional resistance. To address this deviation, a PID control algorithm is introduced to achieve closed-loop speed regulation optimization. Initially, the proportional (P) control function is adopted: the error value is calculated as the target value minus the current actual value, which is then multiplied by the proportional coefficient $k_{P}$ [19]. The resulting output is used to adjust the duty cycle of the motor drive, thereby regulating the motor speed, as shown in Formula (6).

(6) $$\Delta u\left(k\right)=K_{p}\cdot \left[e\left(k\right)-e\left(k-1\right)\right]+K_{i}\cdot T\cdot e\left(k\right)+K_{e}\cdot \frac{\left[e\left(k\right)-2e\left(k-1\right)+e\left(k-2\right)\right]}{T}$$

$\Delta u\left(k\right)$: Increment of the control variable (e.g., change in PWM duty cycle);

e(k) = ω_target − ω(k): Current speed error (target rotational speed—actual rotational speed);

T: Sampling period (unit: seconds, recommended range: 0.001~0.01 s);

K_p_, K_i_, K_e_: Proportional, integral, and derivative gains.

**Figure 7:**
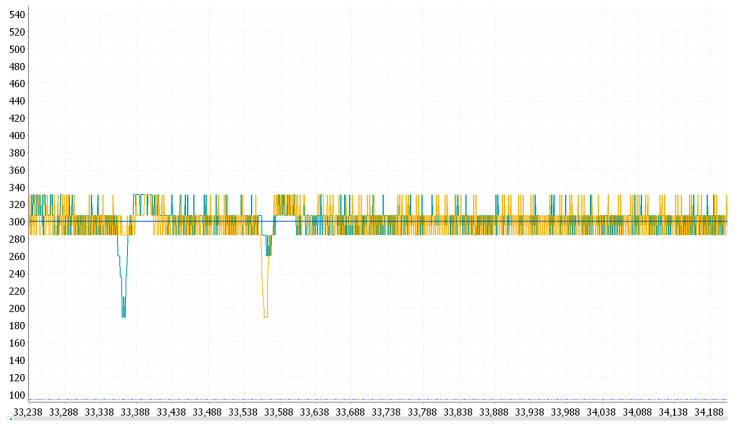
PID control motor waveform: when resistance is applied, the motor can quickly adjust and return to normal.

Next, the proportional-integral (PI) control function is introduced. Based on P control, it accumulates errors over multiple iterations, causing the error to gradually build up over time. This accumulation ultimately breaks the original balance and generates an output, thereby eliminating steady-state errors. Finally, the proportional-integral-derivative (PID) control function combines the effects of P, I, and D. Based on PI control, it calculates the difference between the previous error and the current error, multiplies this difference by the derivative coefficient, and then adds the result to the P and I terms to form the final output [20]. It is only necessary to adjust the values of $k_{P}$, $k_{i}$, and $k_{d}$ according to the waveform until the motor speed can be quickly corrected when resistance is suddenly applied. The PID tuning process is shown in Figure 7.

Compared with the scenario without the PID control algorithm, after incorporating the PID control algorithm, the initial speed of the motor can quickly reach the set target speed. Moreover, when affected by factors such as load changes, voltage fluctuations, or frictional resistance, the motor speed can be quickly corrected, thereby reducing the impact of sudden incidents on speed during actual transportation.

### 3.4. Static Load Stacking Test for Transport Boxes

The transport box used in this paper is made entirely of polyvinylidene fluoride (PVDF), a high-performance fluoropolymer, which is increasingly being applied to high-end transport packaging due to its excellent mechanical properties, chemical corrosion resistance, and thermal stability [9]. Compared with traditional transport packaging materials such as cartons and ordinary plastics, PVDF materials offer higher strength, better toughness, and longer service life, making them particularly suitable for transport scenarios with high requirements for safety and reliability. Moreover, the box design adopted in this paper allows for the customization of different airtight containers based on the specific needs of various laboratories.

To verify the reliability of the selected material, this paper conducted a static load stacking test. The static load stacking test is a key method to evaluate the vertical pressure-bearing capacity of transport packages during storage or transportation. By simulating actual stacking conditions, this test effectively assesses the deformation resistance, load-bearing limit, and stability of shipping containers under constant static loads, providing a scientific basis for packaging design optimization and logistics safety. The transport container used in this test has specifications of 232 × 180 × 140 mm. The static load stacking test for PVDF material transport containers is designed primarily based on the following standards: GB/T 4857.3-2008/ISO 2234:2000 Packaging-Basic Tests for Transport Packages-Part 3: Stacking Tests Using Static Loads [21,22].

The basic formula for calculating the applied force in the static load stacking test of PVDF transport containers is shown in (7).

(7) P=W×k×(n−1)

P: Total force to be applied (unit: Newton, N);

W: Total weight of a single transport container (including PVDF case and internal loads, unit: N);

k: Safety factor of 1.2;

n: Maximum stacking layers of 5.

In ANSYS (version 2024 R2), deformation calculations are based on the core equations of the finite element method:

1. The basic balance equation is shown in Formula (8).

(8) [K]{u}={F}

[K]: Global stiffness matrix, containing material properties and geometric information;

{u}: Node displacement vector;

{F}: Node load vector.

2. The maximum/minimum deformation calculation formula is shown in (9) and (10).

(9) $$u_{max}=\max\left(\sqrt{u_{xi^{†}}^{2}u_{y_{i}}^{2}+u_{z_{i}}^{2}}\right)$$

(10) $$u_{min}=\min\left(\sqrt{u_{xi^{†}}^{2}u_{y_{i}}^{2}+u_{z_{i}}^{2}}\right)$$

∀i∈ Node.

3. The average deformation calculation formula is shown in (11).

(11) $$u_{avg}=\frac{1}{N}\sum_{i=1}^{N}\sqrt{u_{xi^{†}}^{2}u_{y_{i}}^{2}+u_{z_{i}}^{2}}$$

N: Total number of nodes (N = 4648).

**Figure 8:**
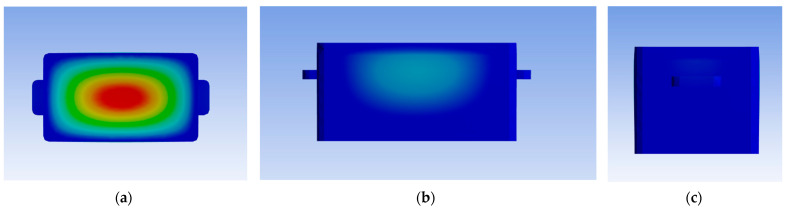
(**a**) illustrates the deformation contour plot of the upper surface of the transport container, (**b**) depicts that of the front face, and (**c**) shows that of the side face. Colors are mapped to pressure magnitude through a gradient of hue, following the heatmap logic of “warm colors representing high pressure, cool colors representing low pressure”.

**Figure 9:**
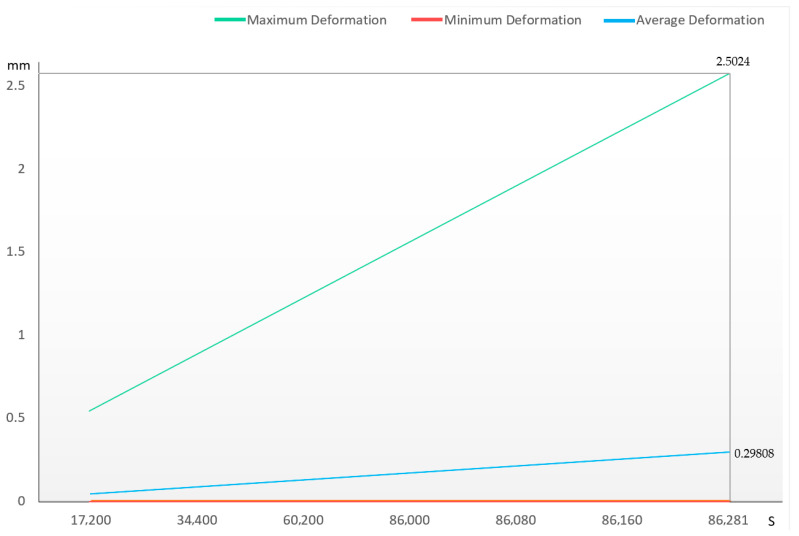
The deformation curve of the transport container versus time. Green is the maximum deformation curve, red is the minimum deformation curve, and blue is the average deformation curve of the object.

A force of 2000 N was applied to the upper surface of the transport container for 24 h. The deformation contour plot is shown in Figure 8, and the deformation curve is shown in Figure 9. The maximum deformation was 2.5024 mm, which is less than 2% of the height for the residual deformation of each face of the container, meeting the standard requirements.

## 4. OpenMV Visual Processing

### 4.1. Device and Working Principle

The OpenMV vision module used in this paper is the model OpenMV4 Cam H7 Plus (OpenMV4 Cam H7 Plus, Xingtong Technology, Shenzhen, China), which is equipped with an STM32H7 processor (STM32H7, STMicroelectronics, Geneva, Switzerland) with a clock frequency of 480 MHz. It has 1 MB of Random Access Memory (RAM) and 2 MB of Flash Memory built in [23]. The module is furnished with an independent control unit, responsible for realizing the tracking function of the image acquisition and processing pan-tilt in the Y-axis direction. The driver module draws power from the power supply, provides the required current for the pan-tilt servo, and thus drives the mechanical components of the pan-tilt to operate. The pan-tilt is based on a self-designed 3D model, assembled into a complete structure via 3D printing and SG90 servos. The aforementioned components form a closed-loop control system, enabling automatic real-time tracking of the target.

The remaining mechanical components of the car include drive motors, an STM32F103C8T6 controller, a transport box, and a support frame. In this system, after identifying target information, the OpenMV vision module controls the pan-tilt servo to achieve Y-axis tracking of the target. Subsequently, the OpenMV vision module transmits the target information to the STM32F103C8T6 controller via serial communication, and the controller drives the motors to complete X-axis tracking of the target. This design has universal applicability: the OpenMV vision module and the pan-tilt servo adopt an independent control architecture, facilitating subsequent researchers to modify the car (e.g., upgrading to a higher-resolution vision module) and thus extending it to a wider range of intelligent application scenarios.

### 4.2. Vision-Based Target Tracking

The OpenMV vision module features open-source, cost-effectiveness, and robust support for machine vision. It integrates OmniVision’s OV5640 CMOS sensor with a resolution of 5 megapixels (2592 × 1944), which can meet the basic needs of daily photography and video recording, and is widely used in fields such as consumer electronics [23,24]. OpenMV adopts the Python programming language, integrates an embedded image sensor and advanced machine vision functions, and can be used as a programmable camera module. The photosensitive element used in this paper has a detachable design, allowing for replacement with a higher-resolution camera according to actual needs.

The OV5640 CMOS sensor is responsible for image acquisition, after which the system invokes its built-in image processing algorithms. This enables the system to calculate the coordinates of the target image’s center point and the field of view, a method widely used in computer vision and image processing. The deviation is obtained by calculating the difference between the coordinates of the target’s center point and the center coordinates of the field of view. This deviation is then used to control the movement speed and direction of the servo in the Y-axis. Specifically, the rotation direction of the servo is determined by the sign (positive or negative) of the deviation, while its movement speed has a linear relationship with the absolute value of the deviation.

The calculation formula of the central coordinate of the target object is as follows (12).

(12) $$x_{c}=\frac{x_{min}+x_{max}}{2},\;\;\;\;\;\;y_{c}=\frac{y_{min}+y_{max}}{2}$$

$x_{min},y_{min}$ is the top-left coordinate of the target bounding box.

$x_{max},y_{max}$ is its bottom-right coordinate, and $x_{c},y_{c}$ represents the center coordinate of the detected target object.

In detection tasks based on pre-trained models, the center coordinates can be directly output by the heatmap layer of the FOMO network architecture. Next, the deviation between the centroid of the target object and the image center is calculated, as shown in Formula (13).

(13) $$\Delta x=x_{c}-x_{0},\;\;\;\;\Delta y=y_{c}-y_{0}$$

Δx and Δy are the deviation values in the horizontal and vertical directions.

To verify the feasibility of deploying target detection models on mobile embedded devices such as the OpenMV4 Cam H7 Plus module, this paper trains target detection models using MobileNetV2 and FOMO MobileNetV2 networks, respectively, and compares their computational load and detection accuracy. FOMO (Fast Object Detection and Tracking), which takes MobileNetV2 as its backbone network, is suitable for lightweight target detection tasks in mobile devices and resource-constrained environments [25]. MobileNetV2 is notably characterized by high efficiency; it adopts depthwise separable convolutions and inverted residual structures to minimize computational cost and memory usage without sacrificing performance [26]. These features make it highly applicable to application scenarios in resource-constrained environments.

### 4.3. Object Detection and the Principle of FOMO MobileNetV2 Network

**Figure 10:**
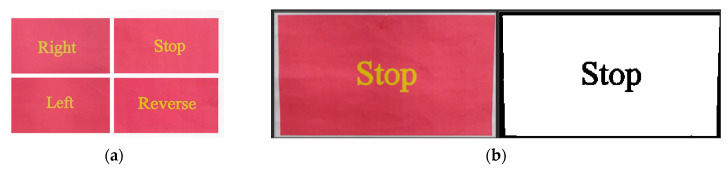
(**a**) shows four kinds of detection objects, and (**b**) shows the color region and semantic map obtained by color segmentation.

In this paper, red markers with a length of 28.5 cm and a width of 16 cm are used as test objects, covering four categories of detection targets, as shown in Figure 10a. Two strategies are adopted during the detection process: when the distance between the car and the marker is greater than 32 cm, the target color region is accurately segmented from the marker image by combining the threshold ranges of H (Hue), S (Saturation), and V (Value), which is used as a visual anchor to approach the target; when the distance is less than 32 cm, deep learning is employed for target recognition to accurately identify the semantic information in the target marker and execute corresponding commands. When the marker has a red background and the command information is yellow, the target features are most prominent after processing by the color segmentation tool of the OpenMV IDE platform, as shown in Figure 10b, which presents the red region and target semantic image obtained by color segmentation of the target picture. In addition, to minimize the impact of variable factors such as environment and illumination on model performance, the training set includes samples under varying lighting conditions and distances to ensure consistency between the training environment and the actual application environment.

The training of MobileNetV2 and FOMO MobileNetV2 network models was conducted on the Python platform. Python (version 3.12.7) is equipped with mainstream deep learning frameworks such as TensorFlow (version 2.19.0) and PyTorch (version 2.2.1+cu118), which provide rich APIs to support the construction, training, and deployment of Convolutional Neural Networks (CNNs). The experimental dataset contains 820 annotated images, with 80% used for model training and 20% for testing. Since FOMO MobileNetV2 0.35 has a larger width multiplier compared to FOMO MobileNetV2 0.1, which can improve detection accuracy while maintaining computational efficiency [27], this paper selects it as the model architecture. To ensure the reliability of results, the MobileNetV2 network model also adopts a width multiplier of 0.35. The model was trained with a learning rate of 0.002 for 200 epochs. Model performance was evaluated using F1-score, Precision, and Recall. During runtime, given that all performance parameters of the OpenMV4 Cam H7 Plus module are known, data such as the system’s per-frame inference time, peak RAM (Random Access Memory) usage, and flash memory usage were obtained through analysis and measurement on the Python platform. After training, the model integrated programs for target recognition, servo control, and data transmission into the execution script via the OpenMV IDE platform and was deployed to the OpenMV4 Cam H7 Plus module through this platform.

Table 2 presents the data changes across each layer (or stage) of the FOMO MobileNetV2 architecture, illustrating the complete process by which data flow from input, through various convolutional layers and specific detection layers, to finally generate a heatmap for target localization. The entire data flow starts with a 256 × 256 RGB input image, first passing through three layers of depth-wise separable convolution (DW Conv) in the MobileNetV2 backbone. At the Truncation point, the network stops further downsampling and retains a high-resolution feature map of 32 × 32. This feature map is fed into the detection head (Head), where the number of channels is compressed to C + 2 (with C being the number of classes), generating a raw Logits output of 32 × 32 × (C + 2). After the Logits are processed by the Sigmoid activation function, the first C channels are converted into probability values within the range [0, 1], while the original values of offsets are retained. A heatmap is generated based on these probability values; finally, through post-processing steps, each position in the 32 × 32 grid is mapped back to the original image coordinates, refined by incorporating the predicted offsets, and filtered via thresholding and non-maximum suppression (NMS) to output the final list of target center coordinates.

The MobileNetV2 network design is based on MobileNetV1 [28]. It retains the simplicity of its predecessor while significantly improving accuracy, enabling state-of-the-art performance in multiple image classification and detection tasks for mobile applications. FOMO MobileNetV2 further embodies these design principles by removing fully connected layers and the final convolutional layer, converting the network output into a compact heatmap that directly encodes the target’s position information and class probabilities. This architectural modification enables FOMO to achieve high performance in object detection tasks with minimal computational requirements [29]. Compared to the standard MobileNetV2, it is more compact and flexible in structure, extremely fast in object detection tasks, and can provide detailed information such as the precise position, size, and quantity of target objects [25]. This is crucial for object detection and tracking tasks, making it a valuable solution for real-time single-object detection and embedded device applications.

**Figure 11:**
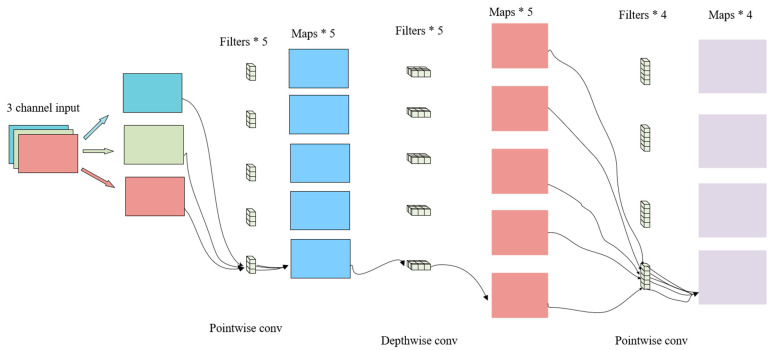
Partial convolution graph of FOMO MobileNetV2.

The basic idea is to replace a full convolutional operator with a factorized version that splits convolution into two separate layers. The first layer is called a depthwise convolution, which performs lightweight filtering by applying a single convolutional filter per input channel. The second layer is a 1 × 1 convolution, called a pointwise convolution, which is responsible for building new features through computing linear combinations of the input channels. The partial convolution of FOMO MobileNetV2 is shown in Figure 11.

**Figure 12:**
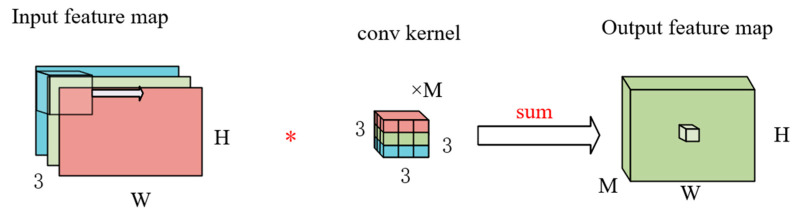
Partial process diagram of the standard convolution diagram.

Standard convolution acts on an input tensor $L_{i}$ with dimensions $h_{i}\cdot w_{i}\cdot d_{i\;}$ (spatial height × spatial width × input channels), applying a convolutional kernel $K\epsilon R^{k\cdot k\cdot d_{i}\cdot d_{j}}$ (spatial kernel size × input channels × output channels) to generate an output tensor $L_{j}$ with dimensions $h_{i}\cdot w_{i}\cdot d_{j}$ (spatial dimensions preserved, channel dimension transformed). The computational cost of standard convolutional layers is $h_{i}\cdot w_{i}\cdot d_{i}\cdot d_{j}\cdot k^{2}$. The partial process of standard convolution is illustrated in Figure 12. This figure depicts the basic workflow of a standard convolution operation: the input is a three-channel feature map (width W, height H), and after the convolution operation, an M-channel feature map is finally generated of size $h_{i}\cdot w_{i}\cdot d_{i}\cdot d_{j}\cdot k^{2}$.

Depthwise separable convolutions can directly replace standard convolutional layers. Empirically, they perform nearly as effectively as regular convolutions, but the calculated cost is as shown in Formula (14).

(14) $$h_{i}\cdot w_{i}\cdot d_{i}\cdot {(k}^{2}+d_{j)}$$

$h_{i}\cdot w_{i}$ accounts for the spatial resolution of the feature map (number of output positions per channel);

$d_{i}$ is the input channels (each contributing to the convolution);

$d_{j}$ is the output channels (each generated by an independent kernel);

$k^{2}$ captures the spatial operations per input channel (multiplications within a single kernel).

This cost represents the sum of the depthwise convolution and the 1×1 pointwise convolution. In practice, depthwise separable convolution cuts down the computation relative to traditional convolutional layers by roughly a factor of $k^{2}$. FOMO MobileNetV2 utilizes k=3 (specifically, 3×3 depthwise separable convolutions), so its computational cost is eight to nine times smaller than that of standard convolutions, with only a slight decline in accuracy [30].

### 4.4. Comparative Analysis of Model Architecture and Technical Characteristics

In this study design, a multi-dimensional visual dataset containing four categories was constructed, with a total of 820 image samples collected under different lighting conditions (80% for the training set and 20% for the test set). The lighting conditions are divided into three typical environments: strong light, weak light, and natural light. Each category is further divided into three gradient intervals by the target distance: short distance (<1 m), medium distance (1–2 m), and long distance (2–3 m), forming a three-dimensional data structure with lighting-distance bivariate variables.

**Figure 13:**
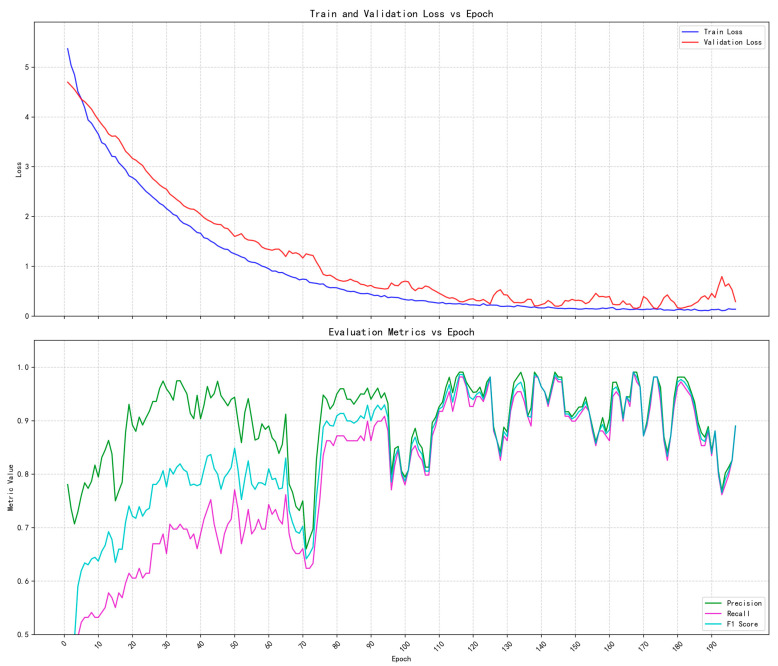
Loss curve and evaluation metrics evolution curve. The figure above shows that the training and verification losses vary with epoch. The following figure shows that the evaluation indicators (precision, recall, F1-score) change with epoch, and the overall curve shows a fluctuating upward trend. The performance of the model on the validation set gradually improves, but the curve “oscillation” shows that the performance is unstable.

During the model training process, a parallel training architecture was adopted to conduct comparative validation between the MobileNetV2 and FOMO MobileNetV2 models. Model optimization and performance evaluation were completed under unified experimental conditions:

1. As a lightweight convolutional neural network, MobileNetV2 has been widely applied in image classification and object detection tasks. Specifically designed for mobile and embedded devices, it aims to balance model accuracy, computational efficiency, and memory footprint. Its core innovations, the Inverted Residual structure and Linear Bottleneck, significantly enhance feature representation capabilities while maintaining efficient inference [25,28]. In the target classification task of this study, MobileNetV2 achieved an average classification accuracy of 67.6% on the test set. The accuracy distribution of each category is shown in Table 3, where the accuracies of categories Left, Right, Stop, and Reverse reached 72.7%, 54.1%, 67.7%, and 75.9%, respectively. The training loss and validation loss tended to converge after the 140th iteration. Due to the limited dataset used in this study for the MobileNetV2 convolutional neural network, the recall, precision, and F1-score did not approach convergence under the same experimental conditions. The evolution of the loss curve and evaluation metrics is shown in Figure 13.

2. The edge computing optimization architecture of the FOMO MobileNetV2 lightweight object detection model, as a real-time visual detection solution for edge devices, constructs a lightweight architecture balancing computational efficiency and detection accuracy through deep integration of the MobileNetV2 backbone network and FOMO (Faster Objects, More Objects) detection algorithm [31]. Based on maintaining the feature extraction capability of MobileNetV2, it enhances the real-time performance of multi-object detection to 2.3 times that of traditional lightweight models.

**Figure 14:**
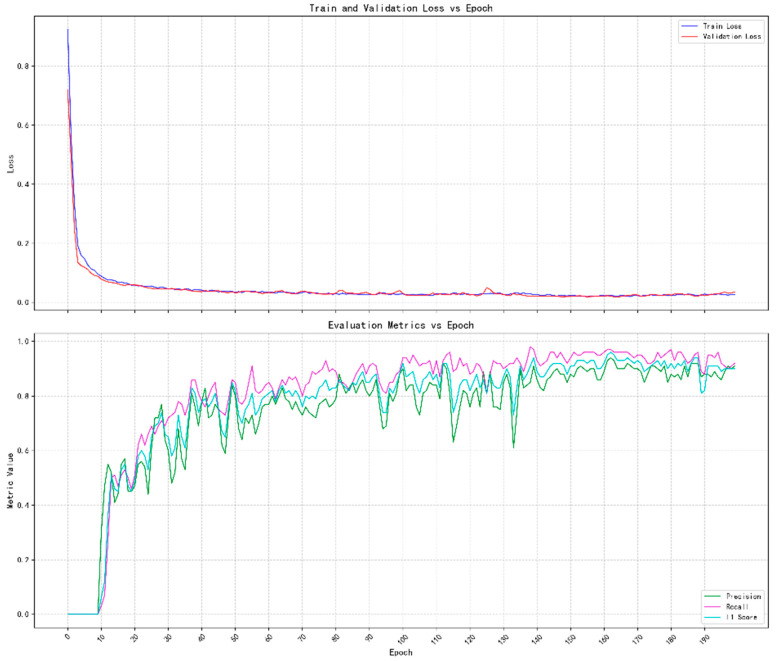
Loss curve and evaluation metrics evolution curve. The early model learning is effective, and the prediction ability is rapidly improved; later indicators tend to be stable, indicating that the model has converged.

FOMO MobileNetV2 achieved an average classification accuracy of 94.1% on the test set. The accuracy distribution of each category is shown in Table 4, where the accuracies of categories Left, Right, Stop, and Reverse reached 93.3%, 94.5%, 93.6%, and 95.1%, respectively. The training loss curve and evaluation index evolution are shown in Figure 14, where the training loss and validation loss tend to converge after the 40th iteration. The recall, precision, and F1-score on the validation set stabilized at 0.91, 0.97, and 0.94, respectively.

**Figure 15:**
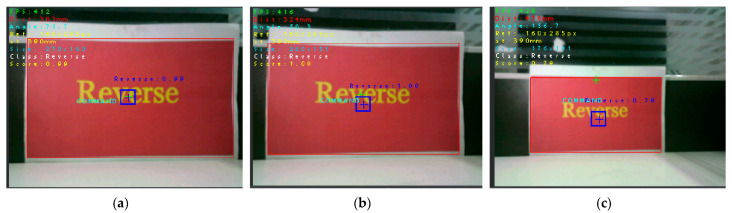
Three target imaging renderings at different distances, where FPS represents the frame rate; Dist indicates the distance from the detection target; Ref represents the imaging size of the real target image at a reference distance of 390 mm; At is the reference distance; Size indicates the length and width of the target image at this time; Class means class; Score indicates accuracy. (**a**) The image shows the effect at a distance of 303 mm, with a model recognition accuracy of 0.99; (**b**) The image shows the effect at a distance of 324 mm, with a model recognition accuracy of 1.00; (**c**) The image shows the effect at a distance of 478 mm, with a model recognition accuracy of 0.79.

Due to the limited resolution of the camera, when the distance to the detection target is relatively large, if target detection is initiated at this time, the accuracy will decrease significantly. To address this issue, this paper conducts real-time detection of targets at different distances via the OpenMV IDE platform. It is found through comparison that when the distance to the detection target is 32 cm, the target imaging effect is the best and the detection result is the most accurate; thus, this paper chooses to start target detection at 32 cm. Figure 15 presents the target imaging effects and corresponding accuracies under three different distances.

The detection results under different lighting conditions are shown in Table 5. Among them, the detection accuracy under normal lighting is the highest, with an average accuracy of 94.44%, followed by that under dim lighting conditions. However, under low-light conditions, the average accuracy is only 13.04%. This is because the camera sensor has limited light-sensing capability, resulting in blurred imaging under insufficient light, which indirectly leads to target detection failure and thus low accuracy. The system designed in this paper is targeted at scenarios with small lighting changes and visibility in the laboratory, so the trained model can meet the requirements.

Experiments show that the model has a Peak RAM usage of 137.7 K and Flash usage of 91.3 K. A program for calculating computational load was added to the detection program on the OpenMV IDE platform, and the computational load was estimated by measuring the ratio of per-frame processing time to total frame time. Practical tests show that if target detection is enabled throughout the entire process, the average load is 83%; while with the two-stage detection mechanism (color threshold segmentation for long distances and lightweight deep learning for short distances), the average load is only 9%, achieving an average inference speed of 55 fps on OpenMV H7 Plus, which is 3.2 times faster than the traditional YOLOv5 Tiny [32]. Experimental verification shows that the system achieves an average recognition accuracy of 94.1% for four types of markers (Forward/Left/Right/Stop). Through experimental comparisons, it was found that FOMO MobileNetV2 achieved the highest average classification accuracy in few-shot object detection, thus confirming that adopting the FOMO MobileNetV2 model meets the design requirements.

### 4.5. OpenMV’s Real-Time Visual Tracking System

This system is a real-time red target tracking and classification recognition system built based on the OpenMV vision module, adopting a modular architecture design. Its functions are collaboratively realized by the hardware control module, visual detection module, and pan-tilt servo module. At the hardware level, it integrates an OpenMV camera, servo pan-tilt, and serial communication module; at the software level, it completes target spatial positioning and category classification by fusing color threshold segmentation with deep learning models, and achieves pan-tilt servo control through a PID control algorithm.

The target spatial coordinates and distance parameters obtained by the OpenMV module are transmitted to the host computer via a serial communication protocol, which executes target tracking and approaching operations based on these data. Achieve high-precision positioning and tracking by integrating target semantic information. Two strategies are used throughout the detection process: when the distance from the cart to the marker is greater than 32 cm, the HSV color space is employed to define the red threshold. Each pixel in the image is compared with the preset color range, where pixels within the range are marked as the target area and others as the background. Since the target is large, it can be recognized and tracked by color even at distances over 300 cm. When the distance is less than 32 cm, deep learning is used for target recognition, which greatly reduces memory occupation.

Target distance estimation is based on the principle of similar triangles, estimating the target distance through the proportional relationship between the spot size and the actual object size [33], as shown in Formula (15).

(15) $$Distance=\frac{Reference\;Distance\times Mean\;Value\;of\;Actual\;Size}{Mean\;Value\;of\;Spot\;Size}$$

**Figure 16:**
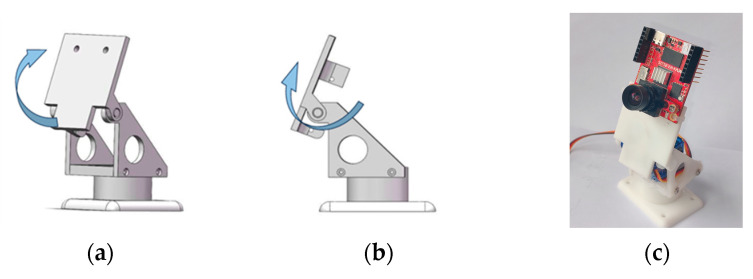
The (**a**,**b**) depict the model diagrams of the pan-tilt; the (**c**) shows the physical diagram of the pan-tilt integrated with the OpenMV module.

In the pan-tilt servo system, the vertical (y-axis) control achieves target centering through a servo motor, which adjusts the pitch angle to maintain the target in the vertical midline of the field of view. For the horizontal (x-axis) direction, the host computer drives the motor to execute target approaching and horizontal centering adjustments, typically by sending pulse width modulation (PWM) commands to align the target with the horizontal central axis. The model and physical diagram of the pan-tilt are shown in Figure 16 below.

**Figure 17:**
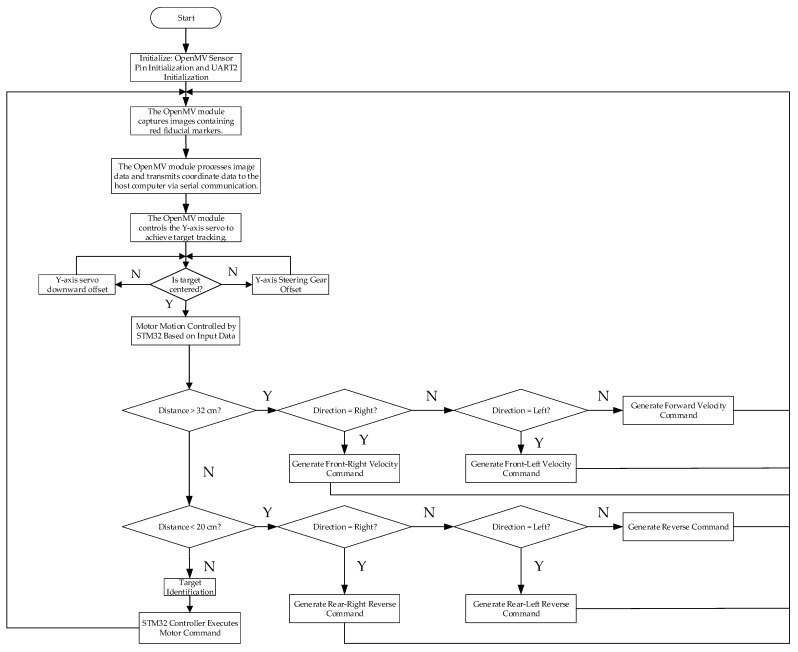
Detailed flowchart of OpenMV target tracking.

The detailed process of target tracking by OpenMV can be referred to in Figure 17, which clearly illustrates the steps and logical relationships from image acquisition to target detection. The system first completes the initialization of the OpenMV camera, servo, and STM32 motor driver; subsequently, OpenMV continuously captures images, identifies the red target in the frame, calculates whether the target is centered, its distance from the camera, and its left–right position, and transmits the information to the STM32 via the serial port; if the target is not centered, the servo drives the pan-tilt to adjust its angle to ensure the target is centered; afterwards, the STM32F103C8T6 microcontroller generates and drives the execution of motor speed and steering commands based on the distance and position, ultimately achieving dynamic tracking of the red target.

## 5. Finished Product Design and Limitation Discussion

In the selection strategy of automobile body materials, acrylic (polymethyl methacrylate, PMMA) sheets are adopted as the main structural material. This material features low density, excellent processing performance, and high impact strength. Meanwhile, its relatively economical material cost also complies with the design’s economic principle. In the structural assembly scheme, the mechanical connection between two acrylic sheets is realized through high-strength stainless steel studs matched with precision lock nuts, ensuring the overall structural rigidity and stability.

The core electronic system of this platform adopts a modular design paradigm. Each module is fixed on the second-layer acrylic base plate through reliable means, and the connection between functional modules is completed via custom-length wires or DuPont wire harnesses. The power management subsystem and key sensing units are all integrated within the first-layer structure. The DC gear motors and infrared sensor pairs adopt a space-height optimized layout scheme to maximize the utilization of the limited internal space volume.

**Figure 18:**
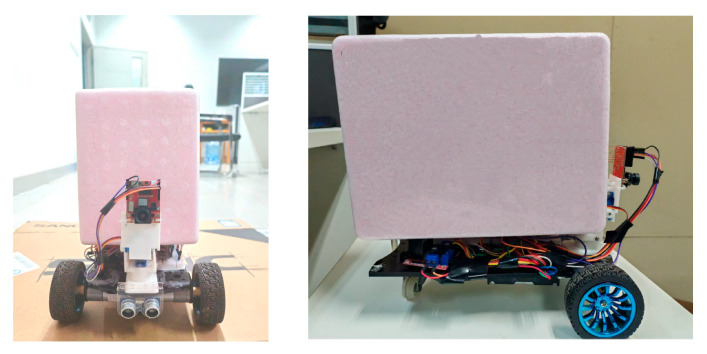
Embedded target recognition car.

The third-tier configuration is a customized sealed functional compartment. This structure integrates a sealed box with excellent wear resistance and chemical corrosion resistance on the basis of the existing automobile platform. The design is highly adaptable, allowing flexible design and integration of different types of closed containers according to the actual needs of diversified experimental scenarios. In this design, a PVDF (polyvinylidene fluoride) material transportation box is used, which is resistant to strong acids, alkalis, and organic solvents, maintains key performance in both low and high-temperature environments, and has lightweight characteristics, providing good protection for laboratory items during transportation [9]. The final design is shown in Figure 18.

Although the system has achieved the expected goals, there is still room for improvement: the accuracy of short-distance target detection has exceeded 90%, but it is susceptible to light interference (e.g., misclassifying “Reverse” as “Right”). In subsequent improvements, more representative detection targets are planned to be adopted. The PID parameters in this paper were adjusted based on experience through iterative tests in a controlled environment; in future work, adaptive or model-based control methods are intended to be used to realize quantitative adjustment of PID parameters under different environmental conditions. In strong backlight or completely dark scenarios, the recognition accuracy of the vision module will decrease significantly, so it is necessary to introduce infrared fill light and dynamic exposure adjustment algorithms. When using the similar triangle principle for distance measurement, system errors usually increase linearly with distance due to factors such as object placement angle, measurement distance, and environmental interference. Therefore, ultrasonic sensors need to be combined for error correction in short-distance scenarios. The floating-point computing capability of STM32F103C8T6 limits the further improvement of model accuracy; in the future, it can be migrated to the STM32H7 series of microcontrollers, while exploring the feasibility of deploying neuromorphic chips. The current power supply cannot support long-term operation, so a wireless charging module needs to be added to extend battery life. The detection targets used in this paper are custom markers with insufficient universality; in the future, markers can be expanded to road signs, indicator signs, etc. Despite the above limitations, the current method can still achieve an average classification accuracy of 94.1% on constrained embedded devices. Finally, when deployed on OpenMV4 Cam H7 Plus, it can reach an average inference speed of 55 frames per second. By communicating with the STM32F103C8T6 microcontroller to control the motor, it can accurately identify the detection target and nearly execute the corresponding commands in practical tests.

## 6. Conclusions

The two-stage detection framework proposed in this study provides a new design idea for embedded vision: through the hierarchical strategy of “coarse positioning and fine recognition”, the adopted model has a peak RAM usage of only 137.7 kilobytes and a Flash usage of 91.3 kilobytes on the STM32H7 processor, reducing computational load by 90%, and can operate reliably on resource-constrained embedded devices. The system successfully achieved dynamic tracking and recognition of red targets in experiments in laboratory transportation scenarios. Such an architecture can be extended to scenarios such as agricultural inspection and warehousing logistics; in the future, custom target markers can also be replaced with road signs, indicator signs, etc., for application in the field of autonomous driving. Future research will deepen exploration in directions such as environmental adaptability, multi-target processing, and integration with digital twins, so as to promote the wide application of intelligent vision in resource-constrained scenarios.

## Figures and Tables

**Table 1:** PID control parameters for MPU6050 yaw movement.

Variable Name	Initial Value	Functional Description
pidMPU6050YawMovement.actual_val	0.0	Current actual angle value measured by MPU6050
pidMPU6050YawMovement.err	0.0	Instantaneous error (target angle—actual angle) for PID calculation
pidMPU6050YawMovement.err_last	0.0	Previous cycle’s error value (used for derivative term calculation)
pidMPU6050YawMovement.err_sum	0.0	Cumulative error sum
pidMPU6050YawMovement.Kp	0.02	Proportional gain coefficient: adjusts control output linearly with current error
pidMPU6050YawMovement.Ki	0	Integral gain coefficient: currently disabled
pidMPU6050YawMovement.Kd	0.1	Derivative gain coefficient: suppresses dynamic overshoot by responding to the error rate of change

**Table 2:** The FOMO MobileNetV2 data flow analysis table (256 × 256 input, Output Stride = 8).

Stage	Module	Input Dim (B × H × W × C)	Output Dim (B × H × W × C)	Key Operations	Functional Description
1	Input	1 × 256 × 256 × 3	-	Image Normalization	RGB input preprocessing
2	DW Conv1	1 × 256 × 256 ×3	1 × 128 × 128 × 32	Depthwise Separable Conv Stride = 2	Initial feature extraction 4× spatial downsampling
3	DW Conv2	1 × 128 × 128 × 32	1 × 64 × 64 × 64	Depthwise Separable Conv Stride = 2	Mid-level feature extraction Additional 2× downsampling
4	DW Conv3	1 × 64 × 64 × 64	1 × 32 × 32 × 128	Depthwise Separable Conv Stride = 2	High-level semantic features Final 2× downsampling (OS = 8)
5	Truncation	1 × 32 × 32 × 128	1 × 32 × 32 × 128	Network truncation	Preserves 32 × 32 resolution Prevents over-abstraction
6	Head	1 × 32 × 32 × 128	1 × 32 × 32 × (C + 2)	1×1 Convolution	Channel compression and fusion Output: class logits + offsets
7	Logits	1 × 32 × 32 × (C + 2)	1 × 32 × 32 × (C + 2)	Raw output	First C channels: class scores Last two channels: (dx, dy) offsets
8	Sigmoid	1 × 32 × 32 × (C + 2)	1 × 32 × 32 × (C + 2)	Sigmoid activation	First C channels → [0, 1] probabilities Offsets remain unchanged
9	Heatmap	1 × 32 × 32 × (C + 2)	1 × 32 × 32 × 1	max(prob) operation	Visual heatmap generation Highlights target center regions
10	Post-process	1 × 32 × 32 × (C + 2)	Object coordinates	Coordinate conversion + NMS	Final localization

**Table 3:** Classification accuracy distribution by category. The row represents the real label, the column represents the predicted label, and the F1-score is the corresponding F1-score of each category (the indicator of comprehensive accuracy and recall rate).

	BACKGROUND	LEFT	REVERSE	RIGHT	STOP
BACKGROUND	100%	0%	0%	0%	0%
LEFT	2.3%	72.7%	10.5%	3.9%	8.6%
REVERSE	8.7%	13.0%	54.1%	18.8%	6.4%
RIGHT	9.4%	2.2%	13.6%	67.7%	7.1%
STOP	5.9%	7.3%	4.3%	6.7%	75.9%
F1-SCORE	0.88	0.74	0.59	0.68	0.76

**Table 4:** Classification accuracy distribution by category. The row represents the real label, the column represents the predicted label, and the F1-score is the corresponding F1-score of each category (the indicator of comprehensive accuracy and recall rate).

	BACKGROUND	LEFT	REVERSE	RIGHT	STOP
BACKGROUND	100%	0%	0%	0%	0%
LEFT	2.8%	93.3%	0%	3.9%	0%
REVERSE	3.7%	2.0%	94.5%	0%	0%
RIGHT	2.5%	0%	3.9%	93.6%	0%
STOP	4.9%	0%	0%	0%	95.1%
F1-SCORE	1.00	0.95	0.98	0.77	0.91

**Table 5:** Table of test results under different lighting conditions, in which the recall rate, accuracy rate, and F1-score on the validation set are used to verify the performance of the model.

Lighting Conditions	Average Accuracy	Precision	Recall	F1-Score
Normal light	94.44%	0.95	1	0.97
Darker light	88.64%	0.95	0.91	0.93
Low light	13.04%	0.32	0.13	0.19

## Data Availability

The dataset is not readily available to the public as it is privately collected and produced.

## References

[1] Qiu C., Tang H., Yang Y. (2024). Machine vision-based autonomous road hazard avoidance system for self-driving vehicles. Sci. Rep..

[2] Hu X., Long C., Bo T., Cao D., He H. (2018). Dynamic Path Planning for Autonomous Driving on Various Roads with Avoidance of Static and Moving Obstacles. Mech. Syst. Signal Process..

[3] Lu Y.W., Tang W.T., Li Y.Z., Wei C.G. (2025). Automatic Cross-Modal Joint Calibration of LiDAR and Cameras. Automot. Pract. Technol..

[4] Zhou Z., Song Z., Cui Y. (2020). Real-time kiwifruit detection in orchard using deep learning on Android^TM^ smartphones for yield estimation. Comput. Electron. Agric..

[5] Liu X.F. (2025). Research on Dynamic Visual SLAM for Mobile Robots Based on Semantic Segmentation and Object Detection. Master’s Thesis.

[6] Li N., Ho C., Lee L. (2022). A Progress Review on Solid-State LiDAR and Nanophotonics-Based LiDAR Sensors. Laser Photonics Rev..

[7] Barbu T. (2011). An Automatic Face Detection System for RGB Images. Int. J. Comput. Commun. Control.

[8] Nie Y.Y., Qi Y.F., Zhan B.R., Li X.Q. (2022). Modular Experimental Application Based on 51 and STM32 Microcontroller. Inf. Comput..

[9] Mehta A., Singh R., Kumar V. (2024). On 3D printed polyvinylidene fluoride-based smart energy storage devices. J. Thermoplast. Compos. Mater..

[10] Park B., Kim S., Su K. (2024). Study on Compensation Method of Encoder Pulse Errors for Permanent Magnet Synchronous Motor Control. Mathematics.

[11] Sharbati R., Rahimi R., Amindavar H. (2020). Detection and extraction of velocity pulses of near-fault ground motions using asymmetric Gaussian chirplet model. Soil Dyn. Earthq. Eng..

[12] Lin S., Nguyen D., Chen H. (2022). Prediction of OLED temperature distribution based on a neural network model. Microsyst. Technol..

[13] Nagy R., Kummer A., Abonyi J., Szalai I. (2024). Machine learning-based soft-sensor development for road quality classification. J. Vib. Control..

[14] Wang H., Wang C. Intelligent Obstacle Avoidance Patrol Car based on Raspberry Pi 4B. Proceedings of the 4th International Symposium on Application of Materials Science and Energy Materials.

[15] Kumar A., Sarangi A., Mani I. (2023). Evaluation of Ultrasonic Sensor for Flow Measurement in Open Channel. J. Sci. Ind. Res..

[16] Adarsh S., Ramachandran K., Nair B. (2022). Improving Range Estimation Accuracy of an Ultrasonic Sensor Using an Adaptive Neuro-Fuzzy Inference System. Int. J. Robot. Autom..

[17] Nadh G., Rahul A. (2022). Clamping Modulation Scheme for Low-Speed Operation of Three-Level Inverter Fed Induction Motor Drive With Reduced CMV. IEEE Trans. Ind. Appl..

[18] Xiang Y., Zhang T., Zhao S., Zhou L., Tian M., Gong H. A PID Tracking Car Based on STM32. Proceedings of the 2022 International Conference on Artificial Life and Robotics (ICARO 2022).

[19] Hu Y., Wang F.Q., Qin L.F., Gong J., Liu B.K. Application of Fuzzy PID Control Algorithm Based on Genetic Self-tuning in Constant Temperature Incubator. Proceedings of the 2020 Chinese Control and Decision Conference (CCDC).

[20] Zhou X., Zhang J., Wu D. (2023). Linear programming-based proportional-integral-derivative control of positive systems. IET Control Theory Appl..

[21] (2008). Packaging—Basic Tests for Transport Packages—Part 3: Stacking Tests Using Static Loads.

[22] Kim Y., Choe H., Cha C., Im G., Jeong J., Yang I. (2000). Influence of Stacking Sequence Conditions on the Characteristics of Impact Collapse using CFRP Thin-Wall Structures. Trans. Korean Soc. Mech. Eng. A.

[23] Li G., Komi S., Berg R. (2025). A Real-Time Vision-Based Adaptive Follow Treadmill for Animal Gait Analysis. Sensors.

[24] Yang X., Zhang Y., Li H. (2022). Design and experimental study of plasma device for accurate contour scanning. Vacuum.

[25] Zhu Q., Zhuang H., Meng R. (2024). A study on expression recognition based on improved mobilenetV2 network. Sci. Rep..

[26] Sandler M., Howard A., Zhu M., Zhmoginov A., Chen L. MobileNetV2: Inverted Residuals and Linear Bottlenecks. Proceedings of the 2018 IEEE/CVF Conference on Computer Vision and Pattern Recognition.

[27] Crupi L., Butera L., Ferrante A., Palossi D. A Deep Learning-based Pest Insect Monitoring System for Ultra-low Power Pocket-sized Drones. Proceedings of the 2024 20th International Conference on Distributed Computing in Smart Systems and the Internet of Things (DCOSS-IoT).

[28] Ogundokun R., Awotunde J., Imoize A. (2024). Deep Transfer Learning Models for Mobile-Based Ocular Disorder Identification on Retinal Images. Comput. Mater. Contin..

[29] Lobur Y., Vonsevych K., Bezugla N. (2025). Spatial identification of manipulable objects for a bionic hand prosthesis. Appl. Comput. Sci..

[30] Ding Y., Huang H., Zhao Y. (2023). A Non-Destructive Method for Identification of Tea Plant Cultivars Based on Deep Learning. Forests.

[31] Novak M., Dolezal P., Prokysek M. (2024). Intelligent inspection probe for monitoring bark beetle activities using embedded IoT real-time object detection. Eng. Sci. Technol. Int. J..

[32] Lawal O. (2023). YOLOv5-LiNet: A lightweight network for fruits instance segmentation. PLoS ONE.

[33] Sun P., Qi X., Zhong R. (2024). A Roadside Precision Monocular Measurement Technology for Vehicle-to-Everything (V2X). Sensors.

